# Evaluation of corneal incision in femtosecond laser-assisted phacoemulsification

**DOI:** 10.1016/j.clinsp.2024.100572

**Published:** 2025-01-27

**Authors:** Guilherme Horta, Newton Kara-Junior, Rogério Horta

**Affiliations:** a Horta Institute, Rio de Janeiro, Brazil; b Universidade de São Paulo, São Paulo, Brazil

**Keywords:** Femtosecond laser, Corneal incision, Cataract surgery, Incision, Length, Healing

## Abstract

**Purpose:**

This study aimed to evaluate the accuracy and quality of healing of main corneal incisions in femtosecond laser procedures in cataract surgery.

**Methods:**

It was a prospective, non-randomized, investigator-masked study. A total of 37 eyes of 37 patients with indication for cataract surgery were separated into two groups in this prospective, nonrandomized study: Femto group, with incisions automated by femtosecond laser (18 eyes), and Phaco group, with incisions made using a keratome (19 eyes). The planned incisions were 2.2 mm wide and 1.65 mm long. The length of the incision and prevalence of endothelial gap, endothelial misalignment, and localized Descemet Membrane Detachment (DMD) were compared.

**Results:**

The mean incision length was statistically higher in the Femto group in two examinations: 1.64 mm ± 0.16 vs. 1.43 mm ± 0.30 (*p* = 0.001) in Exam 1 (between 2 and 4 days) and 1.58 mm ± 0.22 vs. 1.27 mm ± 0.34 (*p* < 0.0001) in Exam 2 (between 1 and 3 months). No eye presented an endothelial gap in Exam 2. The endothelial gap was higher in the Femto group in Exam 1. In the two examinations, endothelial misalignment was lower in the Femto group, whereas the incidence of DMD was lower in the Femto group in the two examinations.

**Conclusions:**

Automated incisions with femtolaser presented a higher mean length and lower DMD prevalence compared to manual incisions with a keratome.

## Highlights

• After cataract surgery, the prevalence rate of Descemet membrane Detachment was significantly higher in the group with keratome incisions (63.2 %) compared to that with femtolaser incisions (22.2 %).

## Introduction

The femtosecond laser procedure in ophthalmology began with the performance of lamellar sections in laser in situ keratomileusis in refractive corneal surgeries.^1^ Subsequently, its use expanded to perform full-thickness sections of the cornea, enabling different corneal transplant formats.^2^

The first cataract surgery using a femtolaser was performed in Hungary in 2008.^3^ Currently, femtolaser is used in facectomy to perform corneal incisions, capsulotomy, and fragmentation of the lens nucleus.^4^, ^5^, ^6^

Femtolaser-assisted cataract surgery has advantages over the conventional technique, including reduced use of ultrasonic energy,^5^^,^^7^ decreased endothelial damage,^4^^,^^5^^,^^7^ reduced corneal edema in the postoperative period,^5^ and performance of capsulotomy^8^ and more precise corneal incisions.^9^^,^^10^

However, the femtolaser procedure has some disadvantages in relation to the conventional technique, including increased prostaglandin levels in the anterior chamber;^5^ high cost;^6^ and positioning of the incisions farther from the limbus, toward the clear cornea, which may increase the risk of astigmatism.^11^

The corneal incision influences the intraocular manipulation during cataract surgery and the patient's visual result.^12^ However, a definitive consensus on the ideal parameters for constructing a high-quality incision has yet to be established.

Anterior Segment Optical Coherence Tomography (AS-OCT) obtains images of the cornea, measures the incision length, and is used to identify complications, such as incomplete sealing, Descemet Membrane Detachment (DMD), and retraction of the corneal endothelial face near the limbus.^10^^,^^13^

This study aimed to evaluate the accuracy and quality of healing of main corneal incisions in femtosecond laser procedure in cataract surgery compared with keratome incisions in the conventional phacoemulsification technique.

## Patients and methods

This prospective, non-randomized, investigator-masked study was approved by the ethics committee of the Universidade de São Paulo, and all patients provided informed consent to participate in the study, following the guidelines established in the Declaration of Helsinki.

All participants were submitted to preoperative ophthalmologic evaluation. A total of 37 eyes of 37 patients with indication for cataract surgery were allocated into two groups: 19 eyes were assigned to the group submitted to incision using a keratome blade and conventional phacoemulsification (Phaco group) and 18 eyes were assigned to the group with incisions, capsulotomy, and fracture of the lens core with femtosecond laser (Femto group).

The study compared the incision length, as well as the prevalence of endothelial gaps, endothelial misalignment, and localized Descemet Membrane Detachment (DMD).

### Surgical technique

An experienced surgeon (R.C.H.) performed all surgeries with topical anesthesia. In the Femto group, the femtosecond laser platform (LensX®, *Alcon Laboratories*, Inc.) was used. Approximately 5 min prior to laser use, a drop of 0.025 %–0.3 % naphazoline hydrochloride and pheniramine maleate (Claril, *Alcon Laboratories*, Inc.) was instilled to decrease subconjunctival bleeding due to eye suction. After sucking the eye, the main and accessory incisions were adjusted toward the clear cornea, anterior to the limbus. The standard configuration of the main incision was as follows: temporal axis, trapezoid tri-planar architecture, 2.2 mm wide on the epithelial surface and 2.3 mm on the endothelial face, 1.65 mm long, and tunnel with entry angle of 70° and exit angle of 90° After completion of the laser, the residual adhesions of the corneal stromal fibers were dissected with a spatula.

In the Phaco group, the 2.2-mm-wide main incision was made with a disposable keratome (Bisturi Medical 2.2 M, *GeeEdge Medical Instrument* Co., Ltd., China), temporally, in a clear cornea. The planned architecture of the incision was biplanar arched, in the clear cornea, with an objective length of 1.65 mm. The definition of the programmed length was based on studies that demonstrated safety in rectangular-shaped incisions, with the tunnel long enough to generate a valvular effect and make it self-sealing, preventing the influx, and reflux of fluid from the anterior chamber.

The surgery proceeded with the traditional phacoemulsification and the *stop-and-chop* technique. In the end, the main and accessory incisions were hydrated with balanced saline, and a sponge (PVA *Eye Spear*, Cenefom Corp., Taiwan) was used to press the anterior flap and check for leakage. In case of a positive Seidel sign, the hydration of the stroma was repeated until the leak was stopped.

### AS-OCT

The images of corneal incisions were obtained by a single masked examiner who used the AS-OCT software (Cirrus, Carl Zeiss Meditec AG). The endothelial gap and DMD appear in the early postoperative period and persist for up to 3 months.^14^ Retraction of the posterior corneal flap at the incision site appears in 33.3 % of the eyes between 2 and 3 wk.^14^ Based on these results, the examination process occurred in two periods: between 2 and 4 days after surgery and between 1 and 3 months after surgery. The objective was to record images with the following characteristics: incision entry and exit site, main incision site with the largest corneal thickness, changes in the endothelial surface of the cut, and DMD.

### Qualitative and quantitative evaluation of AS‑OCT images

The AS-OCT images present a scale of dimensions defined by the equipment itself. The same investigator analyzed the images of each incision after exporting the images to the ImageJ software.

The evaluation of the architecture of the incisions included the incision length, endothelial gap, endothelial misalignment, and localized DMD. The characteristics described are considered indicators of sealing and healing quality.^12^, ^13^, ^14^, ^15^, ^16^, ^17^, ^18^, ^19^

Length was measured by tracing a line from the point of entry of the incision into the epithelium to the point of penetration into the anterior chamber. The comparison of the final measurement with the initially planned architecture is another quality indicator, since the length of the incision influences intraocular manipulation^20^ and the risk of astigmatism.^21^

The endothelial gap is the apposition of the incisional edges of the posterior cornea^13^ and characterizes an incomplete sealing. The longer the length of the incision and the more inclined the angle of entry into the anterior chamber, the larger the size, and area of the endothelial gap.^12^ Another risk factor is low intraocular pressure.^17^^,^^22^

Endothelial misalignment is defined as a misalignment of the edges of the incision in the posterior cornea and may occur by a combination of limbal margin contraction and central tissue thickening,^14^ indicating incomplete remodeling.^14^ High intraocular pressure is significantly related to its presence, as well as stromal hydration.^17^

The main risk factors for localized DMD are as follows: advanced age, preexisting endothelial diseases, prolonged surgical time, hard cataracts, irregular corneal incisions and inadvertent trauma with blunt instruments or phacoemulsification probe, and incisional trauma.^18^ The longer the effective phacoemulsification time, the greater the risk of DMD.^13^

**Fig. 1:**
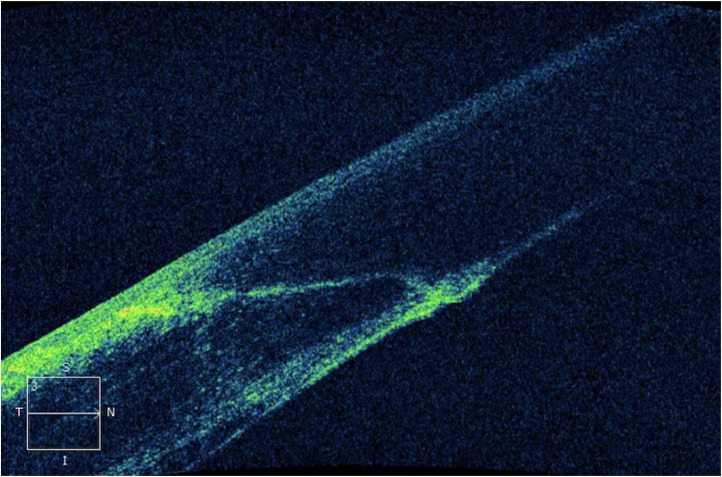
Incision made by femtosecond laser (LensX®, *Alcon Laboratories*, Inc.).

**Fig. 2:**
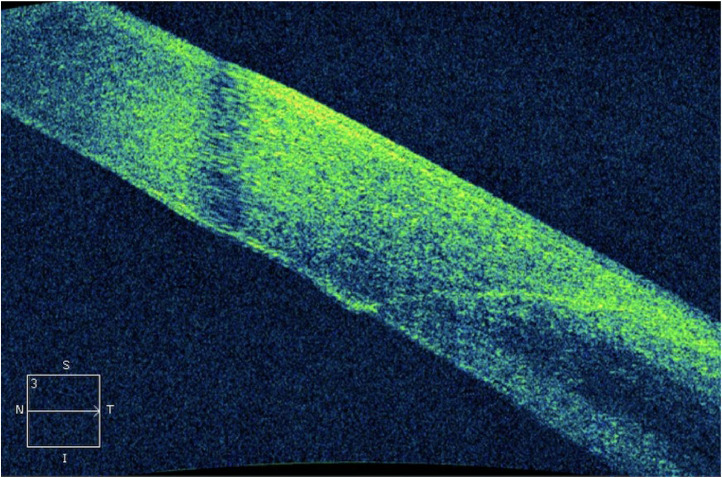
Manual incision with a keratome blade.

**Fig. 3:**
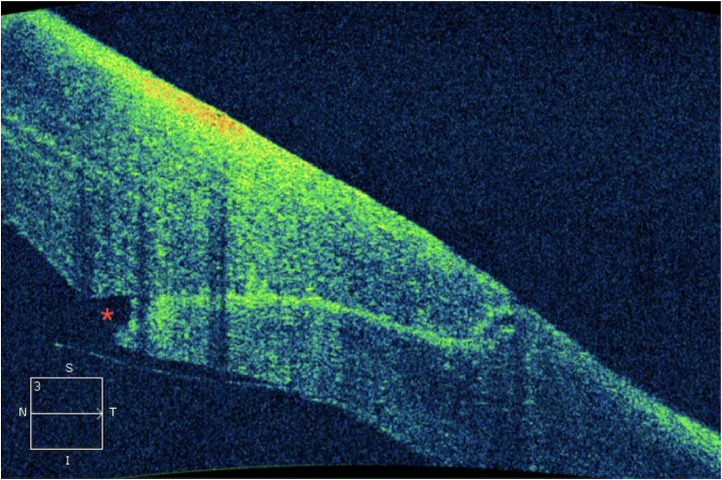
Endothelial gap.

**Fig. 4:**
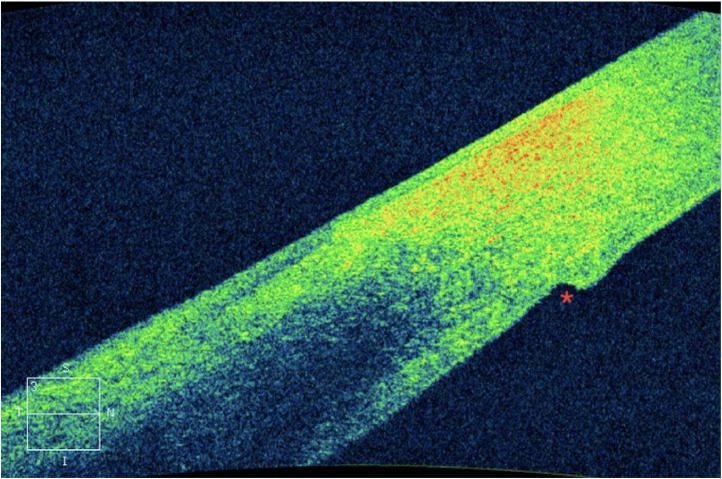
Endothelial misalignment.

Fig. 1 shows an incision made with the femtolaser, and Fig. 2 an incision with a keratome blade. Fig. 3, Fig. 4, and Supplemental Figure 1 illustrate the concepts of endothelial gap, endothelial misalignment, and DMD, respectively.

### Statistical analysis

Descriptive and exploratory data analyses were performed, and means, standard deviations, medians, and minimum, and maximum values were used for quantitative variables and absolute and relative frequencies for categorical variables. Next, the nonparametric Mann–Whitney test was used to compare the groups in relation to the time between surgery and examinations and the length of the incision. The chi-square and Fisher's exact tests were used to analyze the associations of the groups with the presence of endothelial gap, endothelial misalignment, and DMD. All analyses were performed with the aid of the R program, considering a significance level of 5 %.

R Core Team (2023). R: A language and environment for statistical computing. R Foundation for Statistical Computing, Vienna, Austria.

## Results

The study included 37 eyes from 37 patients. Table 1 presents the results of the two groups, conventional phacoemulsification (Phaco group) and femtosecond laser-assisted phacoemulsification (Femto group), in relation to the characteristics of the participants and examinations. There was no significant difference between the groups regarding laterality, sex, and age of the patients (*p* > 0.05).

**Table 1:** Results of the analysis of the sample characterization variables according to the group.

Variable	Category	Group^a^		*p*-value
		Phaco	Femto	
		n (%)		
Sample	‒	19 (100.0 %)	18 (100.0 %)	‒
Eye	Right	12 (63.2 %)	9 (50.0 %)	0.4194^b^
	Left	7 (36.8 %)	9 (50.0 %)	
Sex	Female	14 (73.7 %)	11 (61.1 %)	0.4142^b^
	Male	5 (26.3 %)	7 (38.9 %)	
		Mean (standard deviation; minimum; maximum)		
Age (years)	‒	70.6 (7.0; 58.0; 84.0)	67.8 (7.6; 55.0; 81.0)	0.2875^c^

a Phaco, Conventional Phacoemulsification; Femto, Femtosecond Laser-assisted Phacoemulsification.

b Chi-Square test.

c Mann-Whitney test.

The mean length of the incisions was significantly higher in the Femto group than in the Phaco group (*p* < 0.05) (Table 2 and Supplemental Fig. 2). In the first examination, which was performed in the immediate postoperative period, the mean length of the incisions was 1.64 mm (range 1.35–1.96) in the Femto group and 1.43 mm (range 1.09–2.19) in the Phaco group. In the second examination, at 1 month postoperatively, the mean was 1.58 mm (range 1.27–2.14) in the Femto group and 1.27 mm (range 0.90–2.42) in the Phaco group. In all cases, the length of the incisions, manual, or automated, did not result in intraoperative technical difficulties or complications such as iris prolapse and incisional burn.

**Table 2:** Mean (standard deviation) and median (minimum and maximum value) incision lengths (mm) as a function of the group.

Exam^c^	Group^a^				*p*-value
	Phaco		Femto		
	Mean (standard deviation)	Median (minimum and maximum value)	Mean (standard deviation)	Median (minimum and maximum value)	
Exam 1	1.43 (0.30)	1.36 (1.09‒2.19)	1.64 (0.16)	1.64 (1.35‒1.96)	0.0016^b^
Exam 2	1.27 (0.34)	1.18 (0.90‒2.42)	1.58 (0.22)	1.53 (1.27‒2.14)	<0.0001^b^

a Phaco, Conventional Phacoemulsification; Femto, Femtosecond laser-assisted phacoemulsification.

b Mann-Whitney test.

c Exam 1, AS-OCT between 1 and 4 days after surgery; Exam 2, AS-OCT between 1 and 3 months after surgery.

Table 3 shows that there was no significant association of the type of surgery to which the participants of both groups were submitted, in relation to the presence of endothelial gap and the occurrence of endothelial misalignment (*p* > 0.05). The prevalence of endothelial gaps was 42.1 % and 61.1 % for the Phaco and Femto groups, respectively, in Exam 1, and 0.0 % in both groups in Exam 2. The occurrence rates of endothelial misalignment were 42.1 % and 27.8 % for the Phaco and Femto groups, respectively, in Exam 1, and 31.6 % and 5.6 % for the Phaco and Femto groups, respectively, in Exam 2. In Exam 1, the prevalence rate of DMD was significantly higher in the Phaco group (63.2 %) compared to that in the Femto group (22.2 %) (*p* < 0.05) (Fig. 1). In Exam 2, there was no significant association between the group regarding the presence of DMD (*p* > 0.05), and the prevalence rates were 10.5 % and 0.0 % in the Phaco and Femto groups, respectively.

**Table 3:** Presence of complications: endothelial gap, endothelial misalignment, and Descemet Membrane Detachment (DMD) depending on the group.

Exam^c^	Variable	Group^a^		*p*-value
		Phaco	Femto	
		n (%)		
Exam 1	Endothelial gap	8 (42.1 %)	11 (61.1 %)	0.2476^b^
Exam 2	Endothelial gap	0 (0.0 %)	0 (0.0 %)	‒
Exam 1	Endothelial misalignment	8 (42.1 %)	5 (27.8 %)	0.3615^b^
Exam 2	Endothelial misalignment	6 (31.6 %)	1 (5.6 %)	0.0897^c^
Exam 1	Descemet's membrane detachment	12 (63.2 %)	4 (22.2 %)	0.0120^b^
Exam 2	Descemet's membrane detachment	2 (10.5 %)	0 (0.0 %)	0.4865^c^

a Phaco, Conventional Phacoemulsification; Femto, Femtosecond Laser-Assisted Phacoemulsification.

b Chi-Square test.

c Fisher's exact test. Exam 1, AS-OCT 2–4 days after surgery; Exam 2, AS-OCT 1–3 months after surgery.

Clinical data were also measured in the postoperative period. Significant corneal edema was identified in three eyes in the Phaco group (15.7 %) and three eyes in the Femto group (16.6 %) in Exam 1 (between 2 and 4 days postoperatively). At 1 and 3 months after surgery, the mean best corrected visual acuity was 0.2 logMAR in both groups. The mean keratometry before (Kpre) and after surgery (Kpost) remained stable: Phaco group, Kpre = 42.22 and Kpost = 42.12, and Femto group, Kpre = 42.34 and Kpost = 42.22.

## Discussion

The corneal incision in cataract surgery has been widely studied in the literature and studies that evaluated postoperative results contributed to the understanding of the quality of the incisions. However, there is still no consensus that defines the ideal parameters for its construction.

Visibility^23^ and freedom of intraocular movements,^12^^,^^20^ complete self-sealing that prevents the flow of fluid through the incision^24^ and hypotonia,^25^ and refractive neutrality^21^^,^^26^^,^^27^ can be considered the main qualities of an adequate incision.

### Incision length and architecture

There is no metric definition of a long or short incision in the literature. The presence of the following intraoperative challenges contributes to defining an excessively long incision: difficulty in the mobility of intraocular instruments, decreased visibility due to corneal striae and corneal hydration, and difficulty in the angle of access to cataracts.^20^ About the architecture, the incision is safer when it presents a tunnel with a valve mechanism to prevent the influx and reflux of fluid from the anterior chamber.^24^^,^^28^

Monica and Long demonstrated the safety and efficiency of 3-mm-wide and 2–3-mm-long self-sealing corneal incisions.^29^ Sonmez and Karaca evaluated 2.8-mm-wide rectangular incisions and concluded that there was a statistically greater risk of astigmatism in cases with a length of 1.5 mm than in cases with a length of 1.1 mm.^21^ Conversely, in 2023, Wilczynski et al. ^26^ found no significant difference in the risk of astigmatism between incisions with lengths of 1.4 mm, 1.8 mm, and 2.4 mm.

Ernest et al. evaluated that incisions with a quadrangular shape, that is, equal width and length, are more stable in preventing leaks after external pressure than rectangular incisions.^30^ Masket and Belani presented the safety of quadrangular self-sealing incisions of 2.2 mm and 3.0 mm in preventing leakage and hypotonia in the postoperative period.^25^

In the comparison between the incision models, Grewal et al. reported that the tri-planar architecture was found in only 19 % of the eyes with manual incisions and 100 % of the incisions with femtolaser procedure.^9^

### Incision parameters and evaluation

From the analysis of the data on the efficiency of both rectangular and quadrangular incisions, it is concluded that the planned incisions of 2.2 mm wide and 1.65 mm long, rectangular shape, triplanar with femtolaser, and biplanar with the keratome, used in the present study, present adequate quality parameters.

Stromal hydration at the end of surgery decreases the inflow of fluid into the anterior chamber after surgery.^15^ This technique generates greater local corneal edema^13^ in relation to cases in which hydration was not performed,^16^ and the change in thickness remains for up to 2 wk postoperatively. However, there is no significant difference in the cicatricial aspects of incisions with and without hydration.^16^ Between 5 and 9 wk after an unsutured corneal incision, a cellular reactivation occurs in the stroma, characterizing a final phase of healing.^31^

Therefore, the choice of technique with stromal hydration, along with scheduling the first AS-OCT examination for 2 to 4 days after surgery and the second examination for 1–3 months post-surgery, is appropriate for the analysis of the incisions.

The AS-OCT provides sensitive and detailed measurements of the incision in the clear cornea^13^ and has been widely used for these purposes in the literature. Supplemental Table 1 presents the results of other studies that evaluated the characteristics of tunnel length, endothelial gap, endothelial misalignment, and DMD of major incisions in cataract surgery by means of AS-OCT examinations. Most studies did not analyze the incisions after 1 month, and the authors found in the literature only 3 studies^9^^,^^10^^,^^32^ that evaluated automated incisions with femtolaser. Thus, the evaluation, in the medium term, of incisions 2.2 mm wide and 1.65 mm in length, with the femtolaser platform (LensX®, *Alcon Laboratories*, Inc.) is a novelty of the present study.

The analysis of the causes and consequences of complications of endothelial gap, endothelial misalignment, and DMD has been extensively addressed in other studies.

### Endothelial gap

Calladine and Packard suggested that a longer incision would be less affected by the loss of coaptation because the removal of the margins of the posterior cornea would represent a smaller percentage of the entire incision.^17^ Jin et al. reported a direct and significant relationship between incision length and angle of entry into the anterior chamber with increased endothelial gap area.^12^ The changes in keratometry and spherical equivalent were statistically greater in the cases with endothelial gap.^12^

Bacteria present in the tear can reach the aqueous humor if there is a gap of the inner side of the incision, without needing a complete gap of the incisional tunnel.^14^ In incisions with a 2.75-mm keratome blade, there was a significant increase in anterior corneal astigmatism in the group that presented endothelial gap, at the postoperative period of 1-wk and 1-month, but not after 3-months.^12^ Corneal thickening was statistically greater in patients with endothelial gaps than in patients without it.^13^ The functioning of the corneal endothelial pump could explain the improvement of this alteration over time, as demonstrated by the studies.^12^^,^^14^^,^^16^^,^^33^^,^^34^^,^^35^^,^^36^ Thus, an endothelial gap delays a patient's visual and refractive rehabilitation time.

### Endothelial misalignment

Endothelial misalignment may be caused by the combination of hydration and edema of the incision roof^16^ with retraction of the posterior limbal margin. Wang et al. evaluated endothelial misalignment at 2–3 wk (33.3 %), 1–3-years (75 %), and 3 years (90.5 %) after surgery. The data showed that the prevalence of misalignment increased over the years.^14^ Its clinical effect is unknown, but it is supposed to induce changes in the anterior and posterior curvatures and, consequently, alter the power of the cornea and astigmatism.^14^

### DMD

In cataract surgery, DMD can occur, supposedly, during the construction of the main incision or in the insertion of surgical instruments.^17^ Hypotonia is also correlated with its presence.^17^ DMD impedes the mechanism of the local endothelial pump, which hinders the complete sealing of the incisional wound and, consequently, increases the endothelial gap, and thickening of the cornea at the incision site, leading to slower visual recovery.^18^^,^^19^

### Femtolaser corneal incisions

A study by Chaves et al. found no significant difference between the intended length and that achieved by femtolaser in 1.8-mm tunnel incisions. The authors evaluated the incisions immediately after femtolaser (18 % gap and 0 % DMD) and immediately after completing surgery (91 % gap and 45 % DMD). The results suggest that endothelial gap and DMD are more related to intraoperative manipulation than to laser energy.^10^

Grewal et al. found that endothelial misalignment and DMD were significantly less prevalent in femtolaser-generated incisions than in manual incisions.^9^ The mean length of femtolaser incisions was 1.99 mm (1.86–2.13), which represents 94.9 % of the intended 2.1 mm^9^ and is in line with the result of the current study (95.7 %).

In the present study, two characteristics related to the quality and precision of the incisions were statistically favorable to the femtolaser technique: mean incision length in both exams and lower prevalence of DMD in Exam 1. Conversely, one hypothesis for the higher prevalence rate, although not significant, of an endothelial gap in the Femto group in Exam 1, is the longer mean incision length. There was a higher, albeit not significant, prevalence of endothelial misalignment in the Phaco group, which might be related to the need for more vigorous stromal hydration in this group.

The observation of the cases during the postoperative period suggests that the eyes with DMD presented slower visual recovery, which would constitute a clinical advantage to the use of femtolaser. However, there were no intraoperative complications related to the alterations, and the final visual acuity was similar between the groups. Both surgical techniques showed safe results. Thus, the authors conclude that, although, when submitted to microscopic evaluation, the quality of the incisions may vary according to the technique used, further clinical analysis is still required.

Studies on corneal incisions include different equipment, parameters, and surgical techniques used in both femtolaser and conventional phacoemulsification, as well as diversity in tunnel architecture planning. The advancement of studies on the subject is necessary so that new data help define the currently vague concept of ideal incision.

### Ethical approval

The study was approved by the ethics committee of the Hospital das Clínicas da Faculdade de Medicina da Universidade de São Paulo, and all patients provided informed consent to participate in the study, following the guidelines established in the Declaration of Helsinki.

## Funding

None.

## Declaration of competing interest

The authors declare no conflicts of interest.

## Appendix Supplementary materials

Image, application 1

## References

[1] Nordan L.T., Slade S.G., Baker R.N., Suarez C., Juhasz T., Kurtz R. (2003). Femtosecond laser flap creation for laser in situ keratomileusis: six-month follow-up of initial U.S. clinical series. J Refract Surg.

[2] Farid M., Steinert R.F. (2010). Femtosecond laser-assisted corneal surgery. Curr Opin Ophthalmol.

[3] Nagy Z.Z. (2014). New technology update: femtosecond laser in cataract surgery. Clin Ophthalmol.

[4] Chen L., Hu C., Lin X. (2021). Clinical outcomes and complications between FLACS and conventional phacoemulsification cataract surgery: a PRISMA-compliant meta-analysis of 25 randomized controlled trials. Int J Ophthalmol.

[5] Popovic M., Campos-Möller X., Schlenker M.B., Ahmed I.I. (2016). Efficacy and safety of femtosecond laser-assisted cataract surgery compared with manual cataract surgery: a meta-analysis of 14 567 eyes. Ophthalmology.

[6] Roberts H.W., Day A.C., O'Brart D.P (2020). Femtosecond laser-assisted cataract surgery: a review. Eur J Ophthalmol.

[7] Chen X., Yu Y., Song X., Zhu Y., Wang W., Yao K (2017). Clinical outcomes of femtosecond laser-assisted cataract surgery versus conventional phacoemulsification surgery for hard nuclear cataracts. J Cataract Refract Surg.

[8] Friedman N.J., Palanker D.V., Schuele G. (2011). Femtosecond laser capsulotomy. J Cataract Refract Surg.

[9] Grewal D.S., Basti S. (2014). Comparison of morphologic features of clear corneal incisions created with a femtosecond laser or a keratome. J Cataract Refract Surg.

[10] Chaves M.A.P.D., de Medeiros A.L., Vilar C.M.C. (2019). Architecture evaluation of the main clear corneal incisions in femtosecond laser-assisted cataract surgery by optical coherence tomography imaging. Clin Ophthalmol.

[11] Zhu S., Qu N., Wang W. (2017). Morphologic features and surgically induced astigmatism of femtosecond laser versus manual clear corneal incisions. J Cataract Refract Surg.

[12] Jin K.H., Kim T.G (2019). Relationship between early structural changes at cornea incision sites and surgical outcomes after phacoemulsification. Int J Ophthalmol.

[13] Xia Y., Liu X., Luo L. (2009). Early changes in clear cornea incision after phacoemulsification: an anterior segment optical coherence tomography study. Acta Ophthalmol.

[14] Wang L., Dixit L., Weikert M.P., Jenkins R.B., Koch D.D. (2012). Healing changes in clear corneal cataract incisions evaluated using Fourier-domain optical coherence tomography. J Cataract Refract Surg.

[15] Vasavada A.R., Praveen M.R., Pandita D. (2007). Effect of stromal hydration of clear corneal incisions: quantifying ingress of trypan blue into the anterior chamber after phacoemulsification. J Cataract Refract Surg.

[16] Fukuda S., Kawana K., Yasuno Y., Oshika T. (2011). Wound architecture of clear corneal incision with or without stromal hydration observed with 3-dimensional optical coherence tomography. Am J Ophthalmol.

[17] Calladine D., Packard R. (2007). Clear corneal incision architecture in the immediate postoperative period evaluated using optical coherence tomography. J Cataract Refract Surg.

[18] Singhal D., Sahay P., Goel S., Asif M.I., Maharana P.K., Sharma N. (2020). Descemet membrane detachment. Surv Ophthalmol.

[19] Li S.S., Misra S.L., Wallace H.B., McKelvie J. (2018). Effect of phacoemulsification incision size on incision repair and remodeling: optical coherence tomography assessment. J Cataract Refract Surg.

[20] Menda S.A., Chen M., Naseri A (2012). Technique for shortening a long clear corneal incision. Arch Ophthalmol.

[21] Sonmez S., Karaca C. (2020). The effect of tunnel length and position on postoperative corneal astigmatism: an optical coherence tomographic study. Eur J Ophthalmol.

[22] McDonnell P.J., Taban M., Sarayba M. (2003). Dynamic morphology of clear corneal cataract incisions. Ophthalmology.

[23] Fine I.H. (1994). Clear corneal incisions. Int Ophthalmol Clin.

[24] May W.N., Castro-Combs J., Quinto G.G., Kashiwabuchi R., Gower E.W., Behrens A. (2010). Standardized Seidel test to evaluate different sutureless cataract incision configurations. J Cataract Refract Surg.

[25] Masket S., Belani S. (2007). Proper wound construction to prevent short-term ocular hypotony after clear corneal incision cataract surgery. J Cataract Refract Surg.

[26] Wilczynski M., Kucharczyk-Pospiech M., Omulecki W. (2023). The influence of corneal tunnel length on surgically induced astigmatism after various types of microincision phacoemulsification. Eur J Ophthalmol.

[27] Nagy Z.Z., Dunai A., Kránitz K. (2014). Evaluation of femtosecond laser-assisted and manual clear corneal incisions and their effect on surgically induced astigmatism and higher-order aberrations. J Refract Surg.

[28] Hall G.W., Krischer C., Mobasher B., Rajan S.D. (1993). The construction of sutureless cataract incision and the management of corneal astigmatism. Curr Opin Ophthalmol.

[29] Monica M.L., Long D.A (2005). Nine-year safety with self-sealing corneal tunnel incision in clear cornea cataract surgery. Ophthalmology.

[30] Ernest P.H., Lavery K.T., Kiessling L.A. (1994). Relative strength of scleral corneal and clear corneal incisions constructed in cadaver eyes. J Cataract Refract Surg.

[31] Toto L., Calienno R., Curcio C. (2015). Induced inflammation and apoptosis in femtosecond laser-assisted capsulotomies and manual capsulorhexes: an immunohistochemical study. J Refract Surg.

[32] Mastropasqua L., Toto L., Mastropasqua A. (2014). Femtosecond laser versus manual clear corneal incision in cataract surgery. J Refract Surg.

[33] Dupont-Monod S., Labbé A., Fayol N., Chassignol A., Bourges J.L., Baudouin C. (2009). In vivo architectural analysis of clear corneal incisions using anterior segment optical coherence tomography. J Cataract Refract Surg.

[34] Torres L.F., Saez-Espinola F., Colina J.M. (2006). In vivo architectural analysis of 3.2 mm clear corneal incisions for phacoemulsification using optical coherence tomography. J Cataract Refract Surg.

[35] Can I., Bayhan H.A., Celik H. (2011). Bostancı Ceran B. Anterior segment optical coherence tomography evaluation and comparison of main clear corneal incisions in microcoaxial and biaxial cataract surgery. J Cataract Refract Surg.

[36] Lyles G.W., Cohen K.L., Lam D. (2011). OCT-documented incision features and natural history of clear corneal incisions used for bimanual microincision cataract surgery. Cornea.

