# Supplementary material for: Evaluation of corneal incision in femtosecond laser-assisted phacoemulsification
Source: Clinics (Sao Paulo). 2025 Jan 27;80:100572. doi: 10.1016/j.clinsp.2024.100572 (PMC12013105; doi:10.1016/j.clinsp.2024.100572)

**CLINICS-D-24-01032_Supplementary Material**

**Supplementary Table 1** Summary of studies reporting incision length and prevalence of endothelial gap, endothelial misalignment, and Descemet membrane detachment.

| **Study** | **Eyes** | | **Incision width (mm)** | | **Post-op time** | | **Incision length (mm)** | **Endothelial gap (%)** | | **Endothelial misalignment (%)** | | **DMD (%)** | |
| --- | --- | --- | --- | --- | --- | --- | --- | --- | --- | --- | --- | --- | --- |
| Horta | 31 | | 2.2 | | 2‒4 d | | 1.64 (femto) | 61.1 (femto) | | 27.8 (femto) | | 22.2 (femto) | |
|  |  |  |  |  |  |  | 1.43 (phaco) | 42.1 (phaco) | | 42.1 (phaco) | | 63.2 (phaco) | |
|  |  |  |  |  | 1‒3 m | | 1.58 (femto) | 0 (femto) | | 5.6 (femto) | | 0 (femto) | |
|  |  |  |  |  |  |  | 1.27 (phaco) | 0 (phaco) | | 31.6 (phaco) | | 10.5 (phaco) | |
| Dupont-Monod[33] | 35 | | 2.2 and 2.75 | | 1d | | ND | 49 | | ND | | 51 | |
|  |  |  |  |  | 8d | | ND | 9 | | ND | | 29 | |
| Can[35] | 60 | | 1.8 | | 1d | | 1.67 | 20 | | ND | | 66.6 | |
|  |  |  |  |  | 8d | | 1.52 | 20 | | ND | | 36.6 | |
|  |  |  |  |  | 1m | | 1.43 | 3.3 | | ND | | 3.3 | |
| Torres[34] | 20 | | 3.2 | | 1d | | ND | 25 | | 45 | | ND | |
|  |  |  |  |  | 1m | | ND | 10 | | 15 | | ND | |
| Xia[13] | 60 | | 3.2 | | 1d | | ND | 70 | | ND | | 82 | |
| Calladine[17] | 34 | | 2.5 | | 1hour | | 1.61 | 41 | | 65 | | 62 | |
| Chaves[10] | 11 | | 2.5 | | 1d | | ND | 82 (femto) | | ND | | 36 (femto) | |
|  |  |  |  |  | 1m | | ND | 55 (femto) | | ND | | 0 (femto) | |
| Wang[14] | 113 | | 2.65 and 2.7 | | 1d | | ND | 85.7 | | ND | | 37.1 | |
|  |  |  |  |  | 1‒3m | | ND | 31.8 | | ND | | 4.5 | |
|  |  |  |  |  | 3m | | ND | 3.3 | | ND | | 0 | |
| Grewal[9] | | 36 | | 2.6 | 1m | 1.99 (femto) | | | ND | | 30 (femto) | | 0 (femto) |
|  |  |  |  |  |  | 2.04 (manual) | | | ND | | 38 (manual) | | 18 (manual) |
| Fukuda[16] | | 30 | | 2.4 | 1d | 1.82 | | | 6.7 | | 40 | | 36.7 |
|  |  |  |  |  | 1s |  |  |  | 0 | | 13.3 | | 3.3 |
|  |  |  |  |  | 2s |  |  |  | 0 | | 0 | | 3.3 |
| Mastropasqua [32] | | 60 | | 2.75 | 1s | 1.67 | | | 85.7 (femto) | | ND | | ND |
|  |  |  |  |  | 1m |  |  |  | 36.7 (femto) | | ND | | ND |
| Jin[12] | | 80 | | 2.75 | 1s | 1.67 | | | 70 | | ND | | ND |
|  |  |  |  |  | 1m |  |  |  | 3.8 | | ND | | ND |
|  |  |  |  |  | 3m |  |  |  | 0 | | ND | | ND |
| Lyles[36] | | 25 | | 2.75 | 1d | ND | | | 12 | | 52 | | 4 |
|  |  |  |  |  | 1s | ND | | | 4 | | 60 | | 0 |
|  |  |  |  |  | 1m | ND | | | 4 | | 35 | | 0 |

ND, Not Available; d, Day; s, Week; m, Month.

* 1^st^ Author.

**Supplementary Figure 1** Descemet membrane detachment.

**
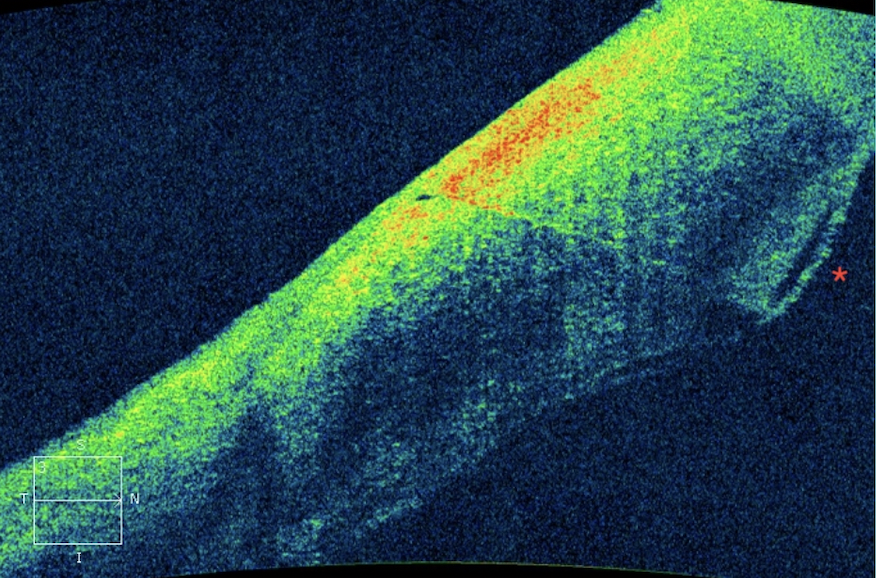
**

**Supplementary Figure 2** Box plot of the incision length (mm) as a function of the group in both examinations. Exam 1, AS-OCT 2–4 days after surgery; Exam 2, AS-OCT 1–3 months after surgery.


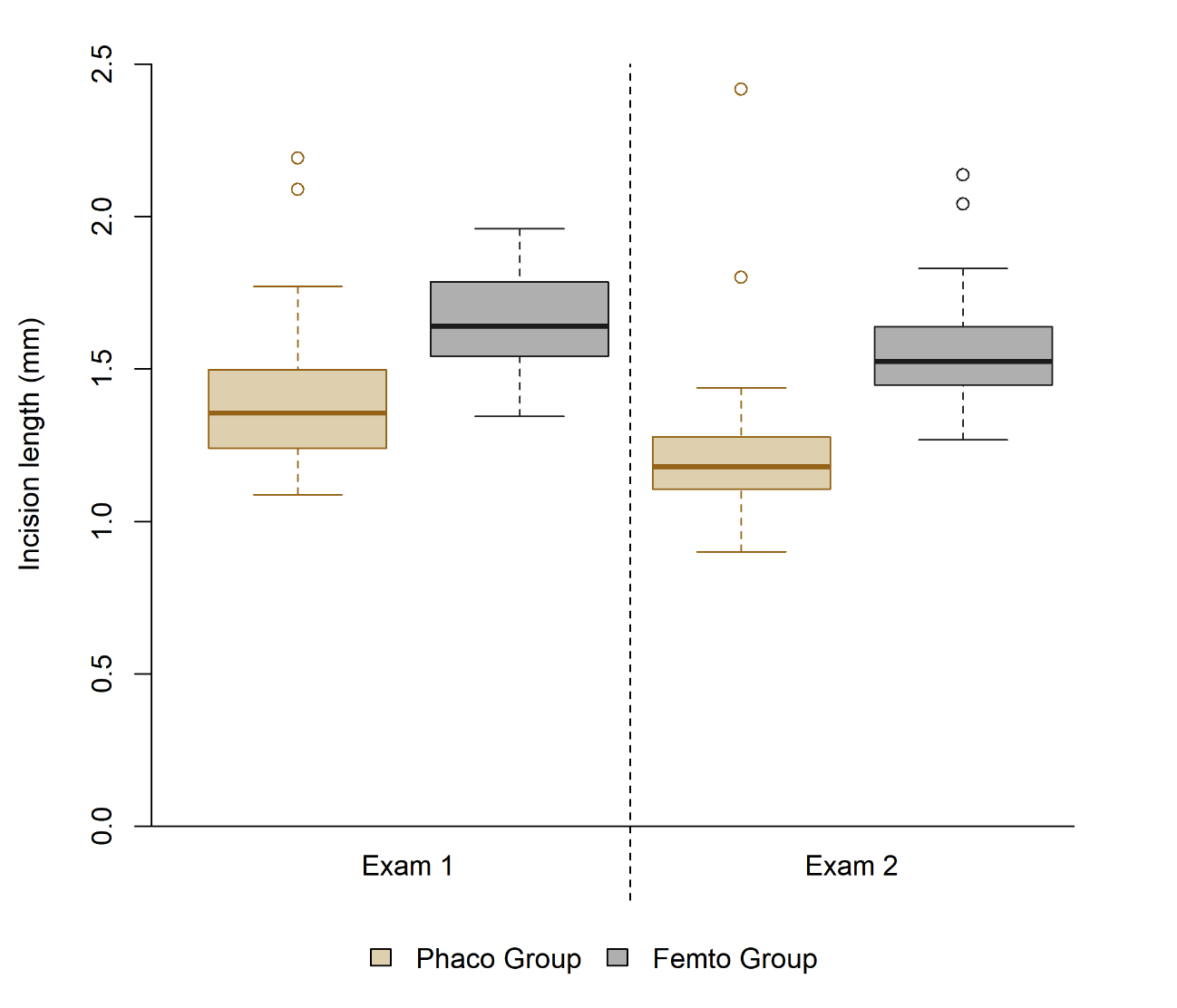

Supplement: Supplementary file 1 [file mmc1.docx]
